# Adsorption and Self-Aggregation of Chiral [5]-Aza[6]helicenes on DNA Architecture: A Molecular Dynamics Study

**DOI:** 10.1021/acs.jpcb.3c02487

**Published:** 2023-09-26

**Authors:** Giuseppina Raffaini

**Affiliations:** † Department of Chemistry, Materials, and Chemical Engineering “Giulio Natta”, Politecnico di Milano, Piazza L. Da Vinci 32, 20131 Milano, Italy; ‡ INSTM, National Consortium of Materials Science and Technology, Local Unit Politecnico di Milano, 20131 Milano, Italy

## Abstract

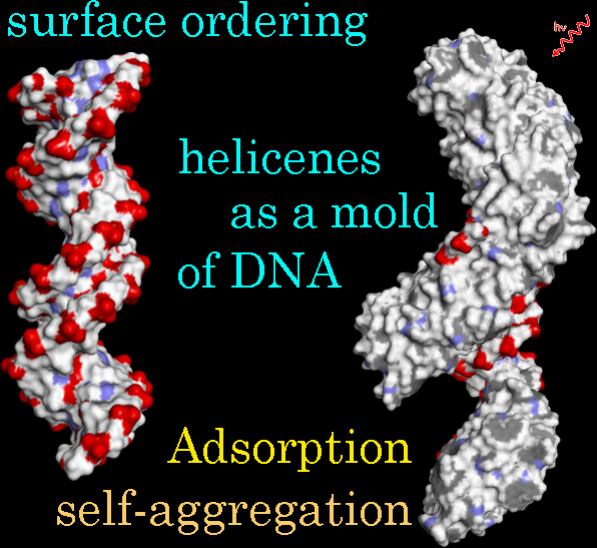

Helicenes are an extremely interesting class of conjugated molecules without asymmetric carbon atoms but with intrinsic chirality. These molecules can interact with double-stranded chiral B-DNA architecture, modifying after their adsorption the hydrophilicity exposed by DNA to the biological environment. They also form ordered structures due to self-aggregation processes with possible different light emissions. Following initial studies based on molecular mechanics (MM) and molecular dynamics (MD) simulations regarding the adsorption and self-aggregation process of 5-aza[5]helicenes on double-stranded B-DNA, this theoretical work investigates the interaction between (*M*)- and (*P*)-5-aza[6]helicenes with double-helix DNA. Initially, the interaction of the pure single enantiomer with DNA is studied. Possible preferential absorption in minor or major grooves can occur. Afterward, the interaction of enantiopure compounds (*M*)- and (*P*)-5-aza[6]helicenes, potentially occurring in a racemic mixture at different concentrations, was investigated, taking into consideration both competitive adsorption on DNA and the possible helicenes’ self-aggregation process. The structural selectivity of DNA binding and the role of helicene concentration in adsorption and the self-aggregation process are interesting. In addition, the ability to form ordered structures on DNA that follow its chiral architecture, thanks to favorable van der Waals intermolecular interactions, is curious.

## 1 Introduction

Helicenes are chiral molecules without asymmetric carbon atoms or other chiral centers in the helical structure.^1,3^ [*n*]Helicenes are polycyclic aromatic compounds formed by *ortho*-fused aromatic rings due to steric repulsive interactions between terminal aromatic rings and chiral helical molecules.^4−9^ It is known that (*M*)-helicenes are levorotatory, while (*P*)-helicenes are dextrorotatory. These molecules have attracted considerable attention due to their interesting electronic and optical properties,^10^ possible applications in asymmetric reactions and catalysis,^11,12^ supramolecular chemistry and molecular recognition biology,^13^ material science,^14^ and molecular motors.^15^ The idea that the extension of the aromatic core of molecular motors, such as the helicene molecule structure, is a viable strategy for red-shifting excitation wavelengths is an interesting concept, as it can be applied to the search for molecular motors driven by visible light.^16^

The synthesis, resolution, and asymmetric synthesis; structural, electronic, and chiroptical properties; and emissions, along with other photochemical properties and applications of helicenes and helicenoids containing main-group elements, such as B, Si, N, and P, either incorporated within the helical backbone or grafted to it, are reported in the literature.^1−3^ The chemistry of heterohelicenes can be traced back to 1903 when the first two azahelicenes were prepared by Meisenheimer and Witte.^7^ The synthesis and properties of helicenes with seven or nine fused aromatic rings are recent developments,^17−23^ together with the enantioselective synthesis, crystal structure, and photophysical/chiroptic properties of aza[10]helicenes with the important indication that the S-form in helicenes increases quantum yields and anisotropy factors but decreases optical rotation values.^24^

Concerning adsorption on chiral double-stranded DNA, helicenes are an interesting new family of molecules with intrinsic chirality that can exhibit chiral selectivity and structural selectivity for binding to DNA. Furthermore, they can discriminate between B- and Z-DNA. Helicenes can both adsorb on biomolecule grooves, leading to the formation of complexes, and intercalate in double-stranded DNA. These aspects are important for the treatment of diseased cells.^25−29^

The adsorption process of [5]-aza[6]helicene, (*M*)-5H, on B-DNA architecture using a theoretical study based on molecular mechanics (MM) and molecular dynamics (MD) methods indicated that the (*P*)-5H enantiomer adsorbs quickly and follows the chiral structure of the helical DNA compared to (*M*)-5H enantiomers, as demonstrated by experiments in which the racemic mixture showed more favorable affinity binding to DNA.^30^ It has been observed that hydrophobic chiral molecules adsorbed on the external surface of DNA can modify the exposed hydrophilic surface by partially modifying its chirality exposed to the biological environment, with consequences for mechanisms involving DNA itself.

In the present work, [5]-aza[6]helicenes are studied, comparing new theoretical results with previous studies that consider [5]-aza[5]helicenes. In the literature, extension of the aromatic core of helicenes is considered an important strategy for red-shifting excitation wavelengths.^16^ This theoretical work focuses on how an extra aromatic ring in the same chiral structure affects the adsorption process on the DNA external surface. These theoretical results regarding [5]-aza[6]helicenes are compared to theoretical results obtained in consideration of [5]-aza[6]helicenes, using the same simulation protocol previously adopted.^30^ For about 25 years, personal attention has been paid to atomistic simulations based on MM and MD methods. The possibility of understanding the role of noncovalent intermolecular interactions is interesting, for example, in the adsorption process of proteins on biomaterial surfaces;^31−34^ in the chiral discrimination in host–guest compounds involving cyclodextrins; and in the nanoaggregation process of carriers and drugs for drug delivery.^35−37^ These theoretical studies can be useful in explaining experimental data from circular dichroism and two-dimensional NMR spectra when the experimental data are accessible. In some cases, these studies may also be useful in predicting the unfolding state and denaturation of albumin on ordered graphite surfaces,^38−40^ the solubilization of carbon nanotubes using proteins^41−43^ or indicating the discrimination of chiral molecules using chiral NTs.^44^

At the atomistic level, the formation, structure, and stability of the layer of chiral [5]-aza[6]helicenes physisorbed on the outer surface of double-stranded B-DNA was investigated, considering enantiopure compounds or racemic mixtures at different enantiomer concentrations as shown in a previous study.^30^

## 2 Materials and Methods

The interaction between 5-aza[6]helicenes and double-stranded DNA was investigated using a theoretical study based on MM and MD methods. The simulation protocol used is the same as that in a previous study on the adsorption of 5-aza[5]helicenes on DNA.^30^ All MM and MD simulations were performed using the consistent valence force field^45^ and the Materials Studio program packages.^46^ The simulation protocol consists of three steps. At first, initial energy minimization of the system is performed. Then, MD runs at a constant temperature are carried out until the equilibrium state is achieved, to study the kinetics of the adsorption process, the mobility on the adsorbed molecule or adsorbed layer, and the self-aggregation process. Finally, the optimization of numerous saved configurations assumed by the system during the MD run as well as the optimization of the geometry assumed by the system at the end of the MD simulations is performed. All MD runs for the study of the adsorption of a single enantiomer of a helicene molecule on a B-DNA chiral structure, and all MD simulations at larger concentrations last 20 ns.^30^ The constant average temperature was equal to 300 K. All simulations were conducted in a dielectric medium considering the distance-dependent dielectric constant of water. The structure of double-stranded B-DNA fragments was found in the Protein Data Bank (3CRO).^47^ B-DNA was always fixed during calculations. The two enantiomers were generated using the Module Builder of the InsightII/Discover program and finally optimized. Using a simulation protocol proposed in previous work,^30,31^ the DNA/5-aza[6]helicene enantiomer interaction was first studied in 1:1 stoichiometry, then at both low and high helicene concentrations, considering a 1:20 stoichiometry in a cubic cell of 123 Å and a 1:120 stoichiometry in a cubic cell of 141 Å, respectively. As in previous work, periodic boundary conditions and initial random arrangements of chiral 5-aza[6]helicene molecules in a simulation box were considered. Therefore, racemic mixtures at both small and larger concentrations were investigated to understand the effect of concentration and competitive adsorption processes on the chiral surface of DNA. Helicene self-aggregation takes place and affects the adsorption process of helicenes’ enantiomers on double-stranded DNA. As such, under the same theoretical conditions and starting with a random arrangement in the same simulation cell but without DNA at both small and larger concentrations, the aggregation process of only the enantiopure (*M*)-6H and (*P*)-6H molecules and their racemic mixtures were investigated. The results of this investigation are reported in the next section.

## 3 Results and Discussion

### 3.1 Chiral 5-Aza[6]helicenes: Self-Aggregation Process at Small and Higher Concentrations of Enantiopure Compounds and Racemic Mixtures

**Figure 1:**
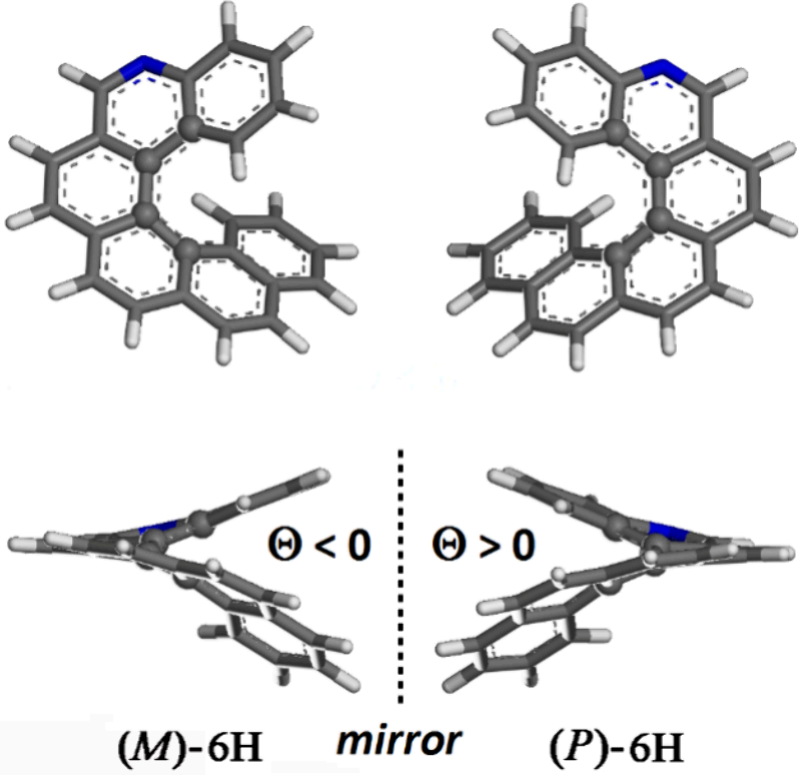
Top view and side view of the optimized geometries of (*M*)-5-aza[6]helicene and (*P*)-5-aza[6]helicene. Color code: carbon atoms are gray, nitrogen atoms are blue, and hydrogen atoms are white.

The optimized geometries of (*M*)- and (*P*)-5-aza[6]helicene single-molecule helicene enantiomers are shown in Figure 1. The dihedral angle Θ for (*M*)-6H is equal to −28.178°, and for the (*P*)-6H enantiomer, the angle is equal to 28.178°.

**Figure 2:**
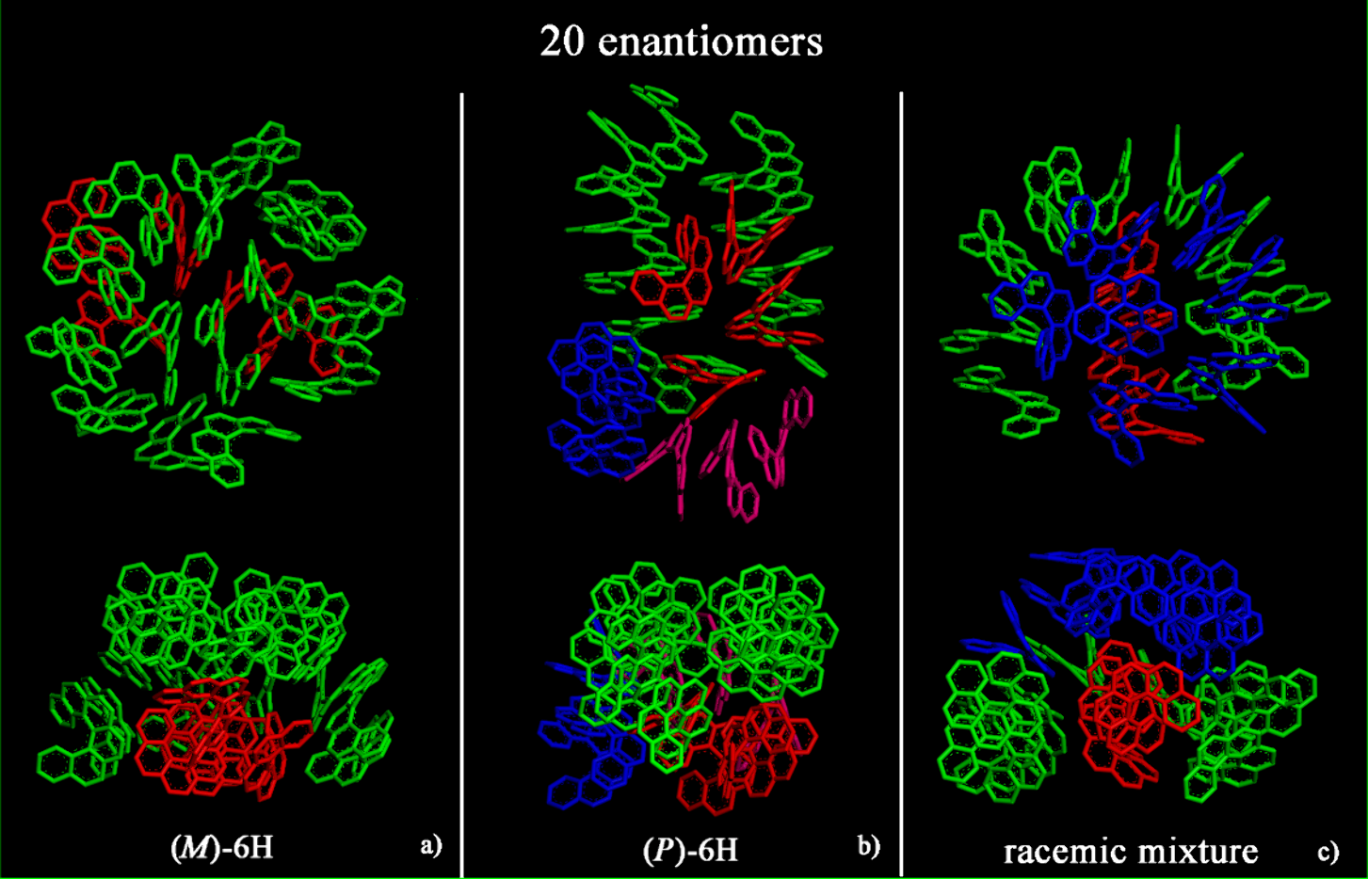
Geometries optimized at the end of MD run lasting 20 ns, considering 20 (*M*)-6H molecules (panel a), 20 (*P*)-6H molecules (panel b), and racemic mixture of 20 enantiomers (panel c). The helicenes with π–π interaction and slightly parallel aromatic rings are colored the same color.

**Figure 3:**
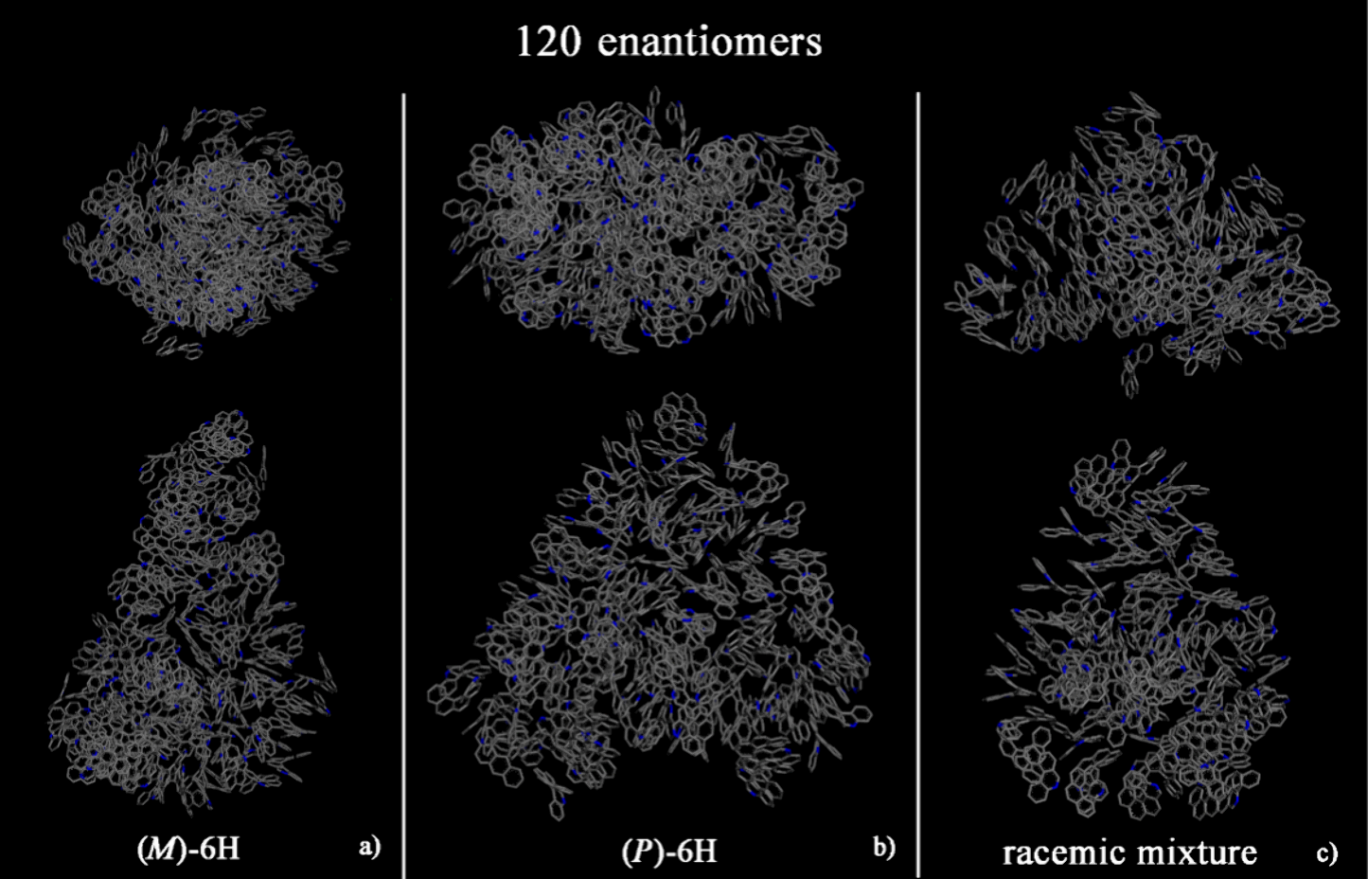
Geometries optimized at the end of MD run lasting 20 ns, considering 120 (*M*)-6H (panel a), 120 (*P*)-6H (panel b), and racemic mixture of 120 enantiomers (panel c). All atoms are in gray, and the hydrogen atoms are omitted for clarity.

Helicenes contain benzene rings, which are interesting building blocks for liquid crystalline systems. These molecules exhibit a nonflat structure in the isolated state and are roughly disk-shaped. Thanks to hydrophobic π–π interactions, helicenes self-aggregate. Using the same simulation protocol proposed in a previous study on the self-aggregation process of (*M*)-5-aza[5]helicene and (*P*)-5-aza[5]helicene, MD simulations at a constant average temperature were performed starting from (*M*)- and (*P*)-6H enantiopure compounds in a racemic mixture in small and higher concentrations initially in a random arrangement on a simulation box (see Figures S1 and S2). At a small concentration (see Figure 2), aggregates were formed during an MD run lasting 20 ns for both the enantiopure compounds (panels a and b) and the racemic mixture (panel c). At a larger concentration (Figure 3), larger aggregates were formed. All animations of the MD runs are reported in SI.

At small concentrations, (*M*)-6H forms more spherical aggregates, while (*P*)-6H displays orientational order along one direction in a helicoidal distribution. A more spherical aggregate is formed in the racemic mixture. At larger concentrations, a conical shape is formed for both the enantiopure compounds and racemic mixture. Hydrophobic π–π interactions and the specific geometry of [6]helicene molecules influence the self-aggregation process.

During the MD run, different aggregates are formed due to the freedom of motion at an average temperature of 300 K, exhibiting similar potential energy. Using this simulation protocol, only the final geometries assumed by the system are optimized; a deeper analysis of all of the geometries of the aggregates assumed by the systems during the MD run is needed to study the different populated aggregates. The helicenes exhibit interesting mobility and different families of aggregates, less or more spherical, at 300 K, which will be reduced after the adsorption process on the DNA surface. This freedom of motion is in fact reduced on the outer surface of DNA, as explained in the following sessions. It is interesting to use solid or biological surfaces to induce aggregation of molecules that can form mesophase or more ordered aggregates,^30^ and to use, for example, different crystallographic surfaces to induce adsorption with one or more ordered layers of molecules parallel and/or perpendicular to the surface^48^ or protein in a specific conformation and tertiary structure to enhance biocompatibility.^34,38^

In this work, how a chiral surface of double-stranded DNA can influence the self-aggregation process of these helicenes or locally induce the adsorption process following its chiral structure is investigated. Just as the different crystallographic faces of TiO_2_ influence the adsorption of small aromatic molecules, DNA structure characterized by its minor and major grooves can influence the adsorption process of [5]-aza[*n*]helicenes, as is to be investigated. After the adsorption process, the helicene molecules are adsorbed on the outer surface of the DNA, following the chiral structure, which shows a positional order in the DNA grooves and orientational order following the DNA chiral structure. Furthermore, thanks to π–π interactions, helicene molecules self-aggregate on the DNA structure, forming aggregates with an ordered arrangement of aromatic rings in minor or major grooves, as has been conducted in previous work.^30^

In the following sections, the theoretical results on the interaction between DNA and (*M*)-6H and (*P*)-6H molecules are reported. The aim of this work is to understand how the chiral surface exposed by minor grooves and major grooves can affect the arrangement of adsorbed molecules or layers of helicenes or self-aggregates attached on the outer DNA surface.

### 3.2 DNA/5-Aza[6]helicene Interaction in 1:1 Stoichiometry: Importance of **Chiral Structure**

**Figure 4:**
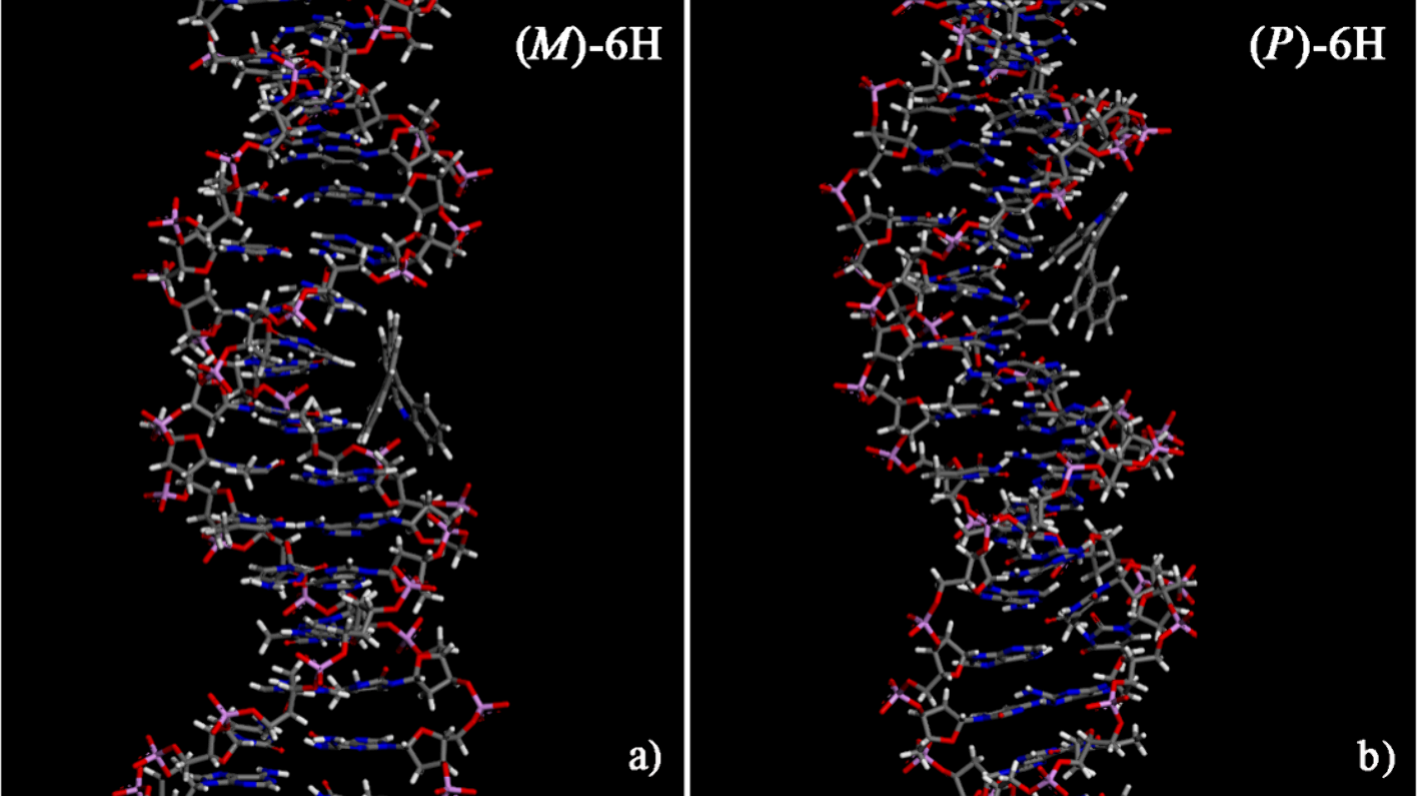
Optimized geometries of (*M*)-5-aza[6]helicene on the left (panel a) and (*P*)-5-aza[6]helicene on the right (panel b) after energy minimization of the conformation assumed by the system at the end of MD run lasting 10 ns. Color code: carbon atoms are gray, nitrogen atoms are blue, oxygen atoms are red, phosphorus atoms are pink, and hydrogen atoms are white.

Initially, using a simulation protocol proposed in a previous study,^30^ the interaction between the minor and major grooves of double-stranded DNA and both (*M*)-5-aza[6]helicene and (*M*)-5-aza[6]helicene enantiomers are studied. The two initial geometries are similar to those reported in Figure S1 of a previous study on [5]helicenes.^30^ In the initial geometries (see Figure S3), individual enantiomers are close to either the minor groove or the major groove of DNA to better understand any chiral discrimination, thanks to favorable interactions at a specific geometric site. After initial geometry optimizations, MD runs of 10 ns and final energy minimizations were conducted. All animations of the MD runs are reported in SI. The stable and optimized geometries calculated at the end of the MD run for two enantiomers near the DNA major groove are shown in Figure 4. The values of the interaction energy, *E*_int_, and the Θ dihedral angle are reported in Table 1. It is interesting to note that during the MD run at 300 K, the two enantiomers interacted with the minor or major groove with very small fluctuations around the site-specific to DNA, indicating a stable and favorable interaction energy and good geometric adhesion.

**Table 1:** Values of the Interaction Energy, *E*_int_, in Kilojoules Per Mole Calculated in the Optimized Geometries Assumed at the End of the MD Run Performed by Two Enantiomers Studied for the Different Final Geometries and the Corresponding Θ Values

site of interaction	(*M*)-HA*E*_int_ (kJ/mol)	Θ value (deg) for (*M*)-6H	(*P*)-HA*E*_int_ (kJ/mol)	Θ value (deg) for (*P*)-6H
minor groove	–167.9	–24.996°	– 181.5	31.857°
major groove	– 184.8	–28.955°	– 192.2	31.366°

The (*P*)-5-aza[6]helicene and (*M*)-5-aza[5]helicene both show more favorable interaction with the DNA major groove, but the (*P*)-6H, rather than (*M*)-6H (see Table 1), does so especially. Interestingly, the (*M*)-5H enantiomer studied in previous studies^30^ showed more favorable interaction strength for the minor groove than the major groove. This fact suggests that a new aromatic ring added to the (*M*)-5H and (*P*)-5H enantiomer modifies the preferential site of interaction and the strength of interaction energy. Concerning the conformational changes after the adsorption process with respect to the isolated [6]helicene molecules, it is interesting to note that the Θ dihedral angles of the (*P*)-6H enantiomer were slightly larger than those in the isolated state. Alternatively, for the (*M*)-6H enantiomer, no significant variations were observed near the major groove, and its Θ dihedral angle is smaller in the DNA minor groove.

### 3.3 DNA/5-Aza[6]helicene Interaction: Importance of the **Concentration**

In this section, the theoretical results regarding the interaction of (*M*)-5-aza[6]helicene and (*P*)-5-aza[6]helicene enantiomers on the chiral surface of double-stranded DNA are reported and discussed considering two different finite concentrations.

#### 3.3.1 DNA/5-Aza[6]helicene Interaction at Small Concentration

**Figure 5:**
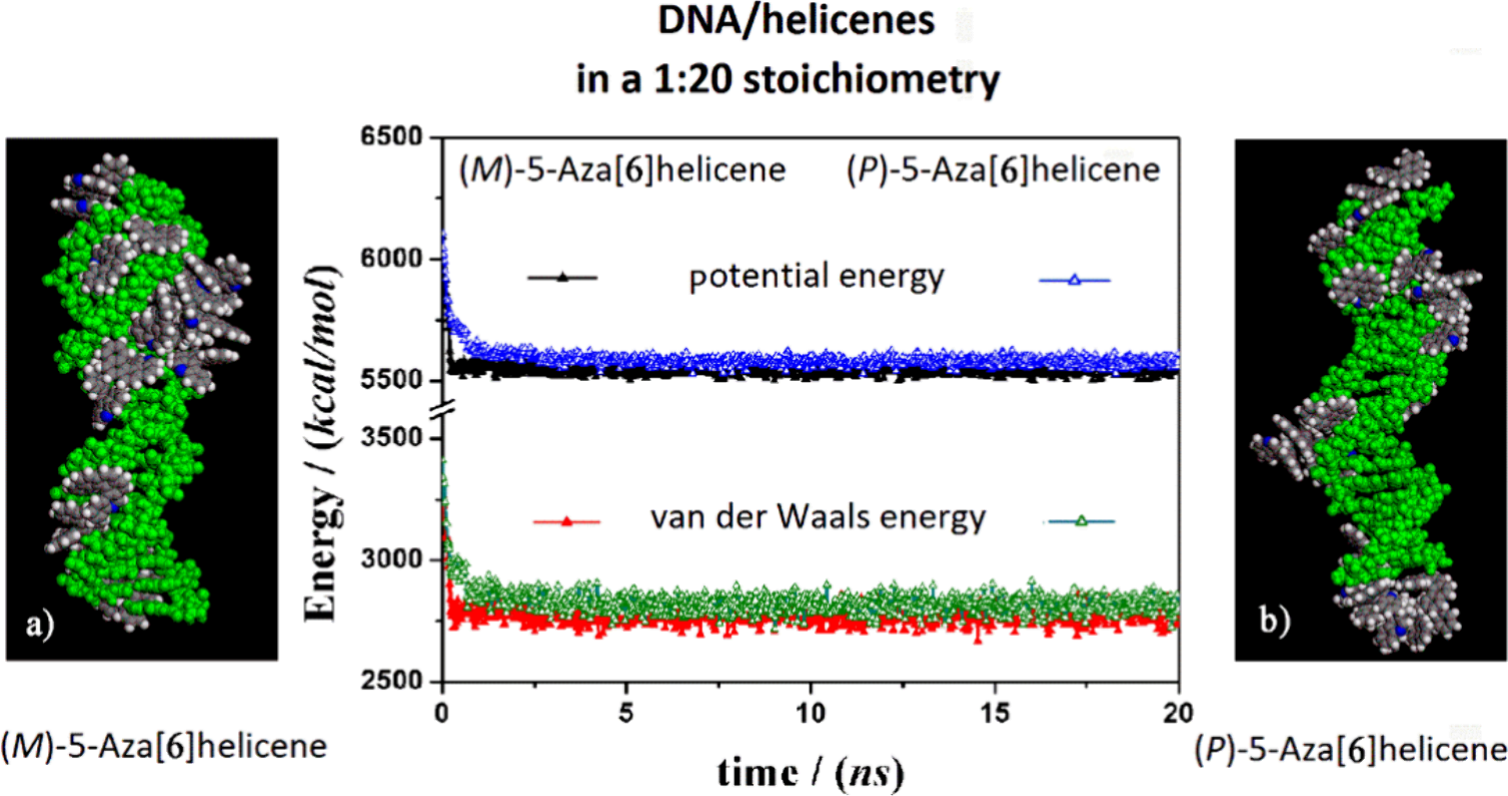
Optimized geometries of *(M*)-6H on the left (panel a) and (*P*)-6H on the right (panel b) after energy minimization of the conformation assumed by the system at the end of MD run considering enantiopure compounds in 1:20 stoichiometry. In the central panel, the potential energy and the van der Waals contribution calculated during the MD runs for the (*M*)-6H (full symbols, black and red) and the (*P*)-6H (empty symbols, blue and green) are reported. All the DNA atoms are in green, and the helicenes have the same color code as shown in Figure 1.

At first, the interaction between B-DNA and chiral enantiopure compounds considered in 1:20 stoichiometry was studied, starting from initial trial geometries and considering a random distribution of (*M*)-5-aza[6]helicene and (*P*)-5-aza[6]helicene molecules around the double strand of the DNA fragment in the center of the simulation box as in previous work (see panel a) in Figures S4 and S2 in ref 30. After the MD run and energy minimization, optimized geometries were obtained, as shown in Figure 5. Specifically, the left panel (panel a) presents the (*M*)-H enantiomer and the right panel (panel b) presents the (*P*)-H enantiomer [6]helicenes. During the MD run, the kinetics of the adsorption process on the outer surface of DNA was very fast for the two different enantiomers and particularly for (*M*)-6H. All animations of the MD runs are reported in the SI. After adsorption on the DNA surface, the helicene enantiomers adhered well on the chiral surface. The adsorbed first layer fluctuated around equilibrium positions. The other [6]helicene molecules interacted via π–π interactions with each other, and they moved slightly on the hydrophobic layer of the first adsorbed helicene molecules. For the two different MD simulations considering two different enantiomers, the values of potential energy and van der Waals contributions reported in Figure 5 (central panel in Figure 5) displayed different kinetics of the adsorption process, which were faster for the (*M*)-6H enantiomer. It should be highlighted that for the same helicenes, i.e., (*M*)-5H and (*M*)-5H, the kinetics of the adsorption process were very similar,^30^ still emphasizing how another aromatic ring in the helicene structure affects the kinetics of the adsorption process on DNA.

During the MD run, the (*M*)-5-aza[6]helicenes at a small concentration displayed more favorable van der Waals contributions and, hence, more stability, as indicated by potential energy calculated during the MD run compared to the (*P*)-5-aza[6]helicene. Conversely, considering the enantiopure (*M*)-5H and (*P*)-5H under the same conditions, similar kinetics processes and stabilities were calculated for two different enantiomers with five aromatic rings. In the final optimized geometries, it is interesting to note that the interaction geometries are slightly different for two enantiomers (see panels a and b in Figure 5). In fact, in the final optimized geometry of the (*M*)-6H enantiomer, more molecules adhered to the major groove, thanks to more favorable interactions, as calculated for a single adsorbed molecule. (*P*)-6H exhibits adsorption in both the minor and the major grooves, displaying favorable interactions for both interaction sites and good interaction with the DNA bases at the end of the DNA fragments. Here, self-aggregation of (*P*)-6H takes place due to favorable interactions between the helicenes and DNA bases.

**Figure 6:**
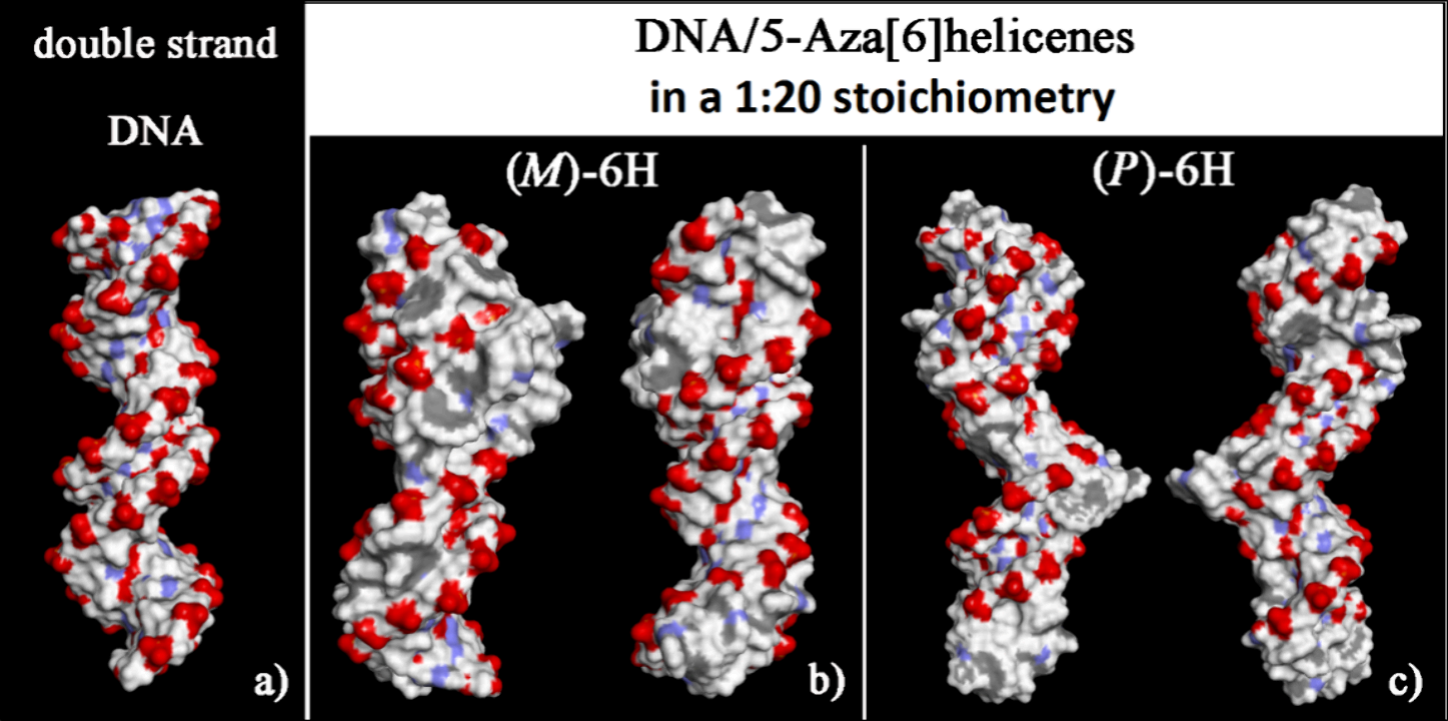
Solvent-accessible surface area (SASA) colored atoms for the optimized geometries of double-stranded DNA from data from the Protein Data Bank on the left (panel a) of *(M*)-6H (panel b) and (*P*)-6H on the right (panel c) in optimized geometries obtained after the MD run, considering enantiopure compounds in 1:20 stoichiometry in each panel from two different viewpoints. Atoms that are partially negatively charged are red, partially positively charged atoms are blue, and apolar groups of atoms are white.

Interestingly, considering the solvent-accessible surface area (SASA) colored by the charge of atoms exposed to the solvent for the two optimized geometries (see Figure 6), it is possible to observe that these new noncovalent DNA/helicene complexes show a hydrophobic patch where the helicenes have adhered to the helical surface.

#### 3.3.2 DNA/5-Aza[6]helicene Interaction at Higher Concentration

**Figure 7:**
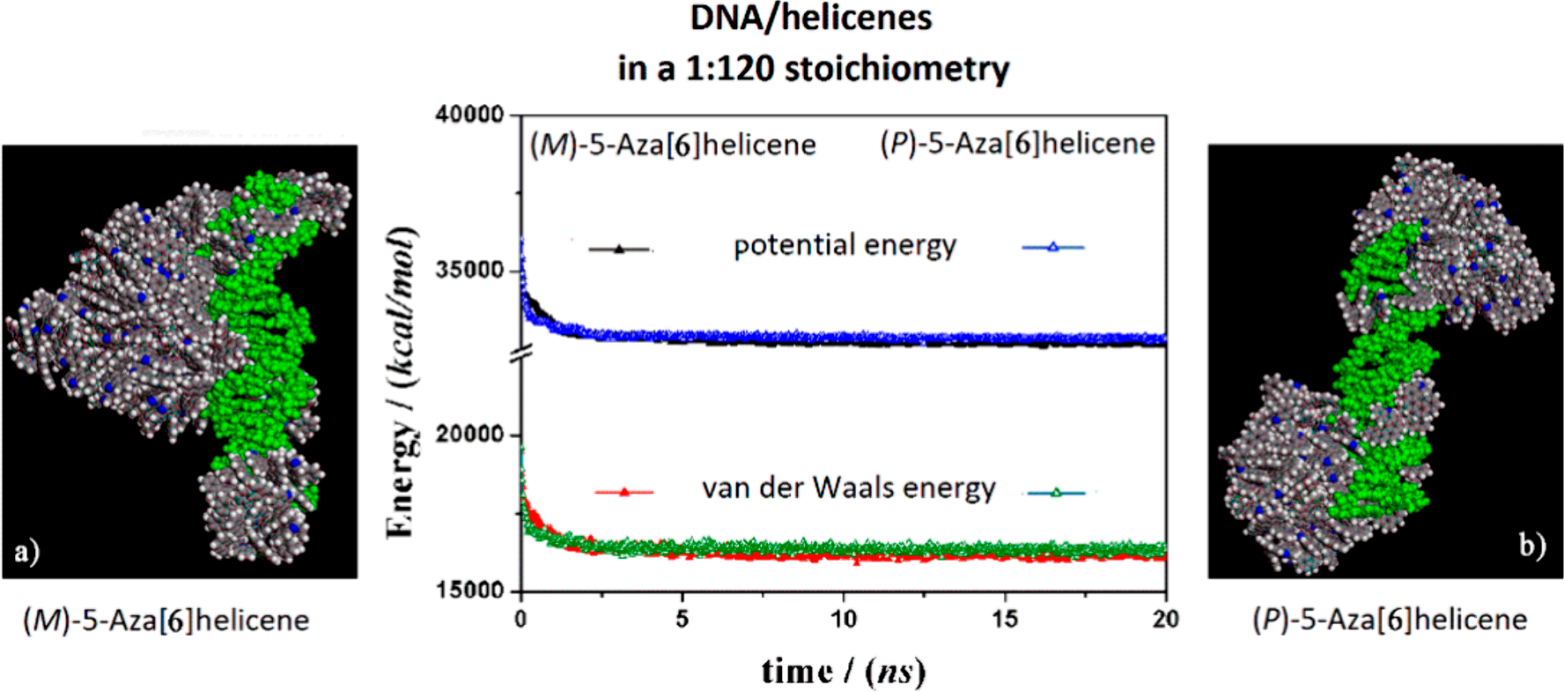
Optimized geometries of (*M*)-6H on the left (panel a) and (*P*)-6H on the right (panel b) after energy minimization of the conformation assumed by the systems at the end of MD run, considering enantiopure compounds in 1:120 stoichiometry. In the central panel, the potential energy and the van der Waals contribution are shown, which were calculated during MD runs lasting 20 ns for (*M*)-6H (full symbols, black and red) and (*P*)-6H (empty symbols, black and red). The color code is the same as in Figure 5.

The interaction between double-stranded DNA and the chiral (*M*)-5-aza[6]helicene and (*P*)-5-aza[6]helicene enantiopure compounds in 1:120 stoichiometries is then considered. Starting from initial trial geometries in a simulation cell as reported in Figure S2 of ref 30 and in panel (b) in Figure S4, and following the MD run and energy minimization, the optimized geometries are reported in Figure 7 for the (*M*)-6H and (*P*)-6H helicenes molecules on the left (panel a) and on the right (panel b), respectively.

Considering both the (*M*)-6H and the (*P*)-6H enantiomers at the highest concentration at the end of the MD run, a similar stability of the system during the MD run was calculated. All animations of the MD runs are reported in the SI. Interestingly, unlike what happened at a smaller concentration, the van der Waals contribution decreased slightly faster for (*P*)-6H with respect to (*M*)-6H (see the central panel in Figure 7). In these MD runs, the adsorption process, as well as the self-aggregation process, also took place before the final adsorption on the DNA surface (see the animations in SI). In fact, some helicene molecules adsorb on the DNA structure, while others self-aggregate in the simulation box and then move to adhere to the DNA surface during MD simulation. Only a few helicene molecules formed a monolayer adherent to the minor groove of (*M*)-6H and on the major groove of (*P*)-6H. As for 5-aza[5]helicenes, (see ref (30)), at a higher concentration, the (*P*)-6H enantiomers kinetically adsorbed faster than the (*M*)-6H molecules (see central panel of Figure 7). Double-stranded DNA acted as a nucleation center for the formation of hydrophobic aggregates by enveloping the DNA structure or starting from the ends of the structure.

It is important to study both the adsorption on the DNA surface and the self-aggregation process of chiral molecules. Different concentrations of helicenes and, therefore, different concentrations of aggregation, or only the adsorption process on the DNA structure at lower concentrations, can influence interactions with light and the kinetics of the adsorption process. They can likely also influence emission properties, depending on the geometry (likely a small part of the mesophase) and the stability of aggregates.

**Figure 8:**
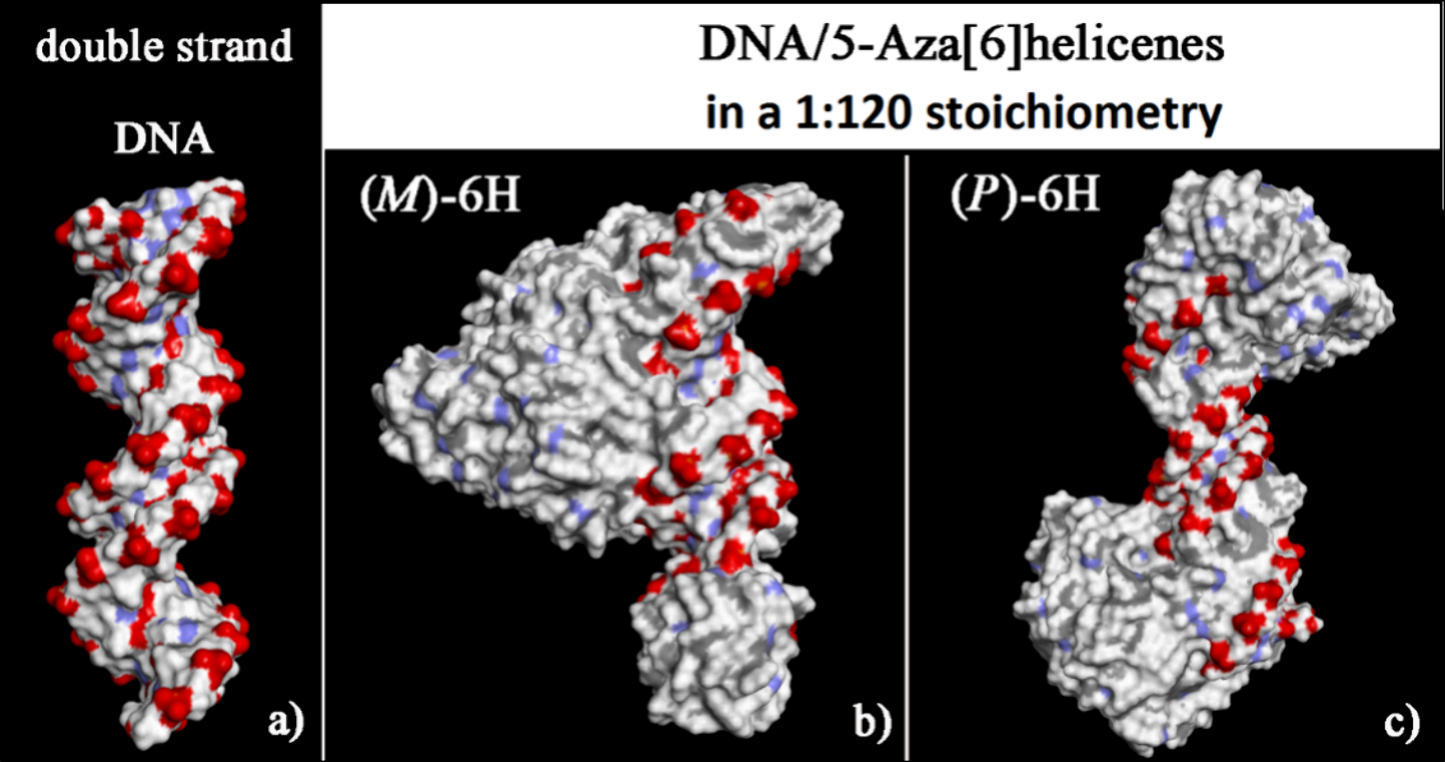
Solvent-accessible surface area colored by atom charge of the optimized geometries of double-stranded DNA from data from the Protein Data Bank on the left (panel a) of *(M*)-6H (panel b) and (*P*)-6H on the right (panel c), after energy minimization of the conformation assumed by the system at the end of the MD run considering enantiopure compounds in 1:120 stoichiometry. The color code is the same as in Figure 6.

The SASA of the optimized geometries after the MD runs is reported in Figure 8. Adsorbed or self-aggregated helicenes on the chiral DNA structure hide the specific helical geometry of oxygen atoms exposed to the biological environment.

### 3.4 DNA/5-Aza[5]helicene Interaction in **Racemic Mistures** at Two Different **Concentrations**

In this section, the theoretical results on the interaction of the (*M*)-5-aza[6]helicene and (*P*)-5-aza[6]helicene molecules in racemic mixtures with the external surface of the chiral DNA at two different concentrations, namely, a small concentration and a higher concentration, are reported. The initial nonoptimized geometries with helicene enantiomers in the initial random arrangement around the double-helix DNA in the simulation box are reported in panel (a) and in panel (b) of Figure S5. All animations of the MD runs are reported in the SI.

**Figure 9:**
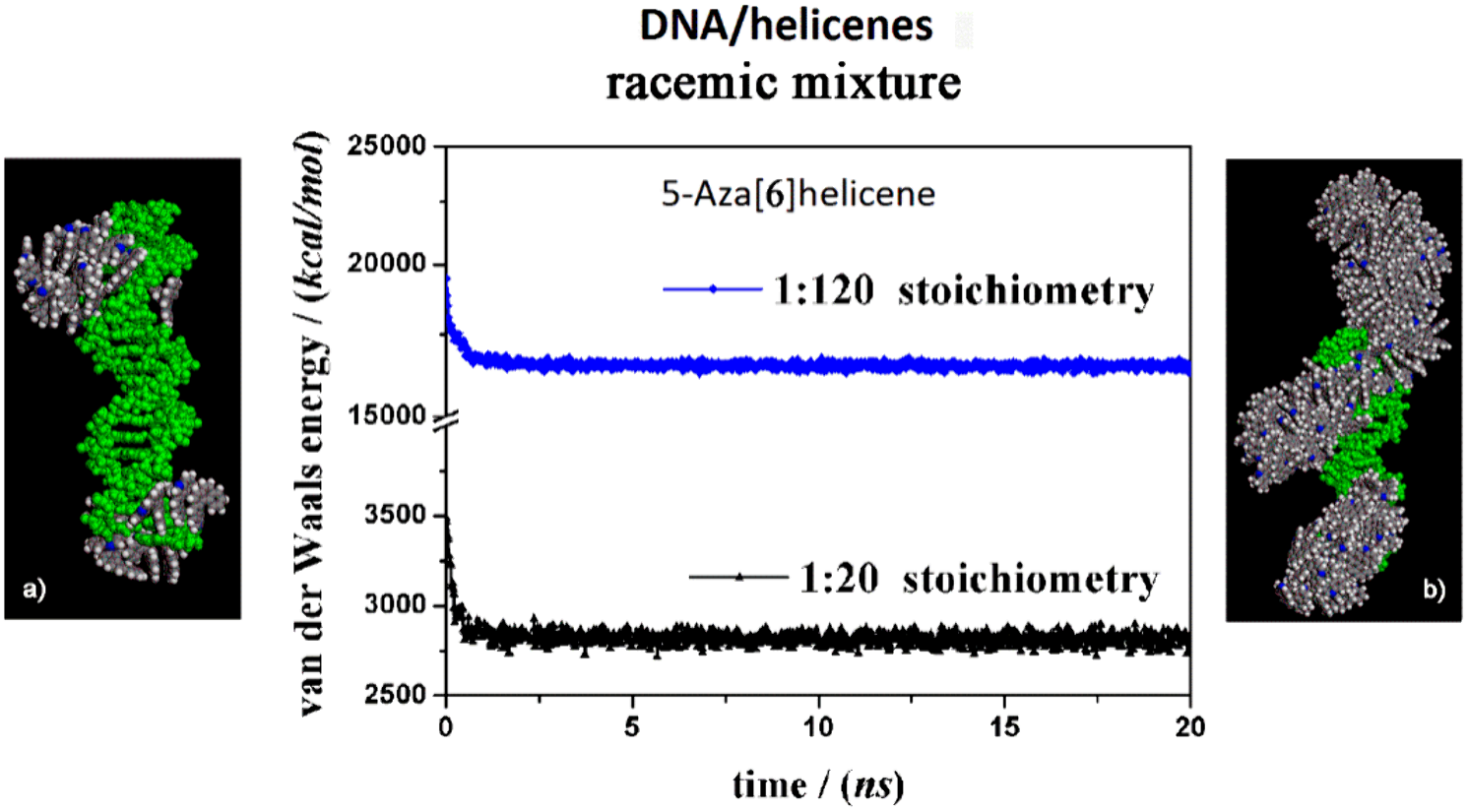
Optimized geometries of (*M*)-6H and (*P*)-6H in a racemic mixture at small concentrations on the left (panel a) and at larger concentrations on the right (panel b) after energy minimization of the conformation assumed by the system at the end of the MD run, considering racemic mixtures, in stoichiometry 1:20 and 1:120, respectively. In the central panel, the potential energy calculated during the MD run lasting 20 ns for the racemic mixture in 1:20 stoichiometry (black symbols) and 1:120 stoichiometry (blue symbols) is shown. The color code is the same as in Figure 5.

Using a simulation protocol proposed in a previous study,^30^ it is curious to note that at a low concentration of racemic mixtures after the MD simulations, all the molecules were adsorbed at the end of the DNA structure, forming an initial layer and small aggregates, thanks to π–π interactions between aromatic rings (see panel a in Figure 9). On the contrary, considering (*M*)-5H and (*P*)-5H in previous work,^30^ the racemic mixture favored interaction with the DNA fragment with a monolayer of molecules that follow the helical structure, particularly the major groove, which was similar to a glove. At a higher concentration (see panel b in Figure 9), the helicene molecules formed a layer in the major groove, and the aggregation of helicene molecules occurred at the end of the DNA structure. Initially, a layer of helicene molecules was adsorbed onto the DNA surface within 5 ns, at both 1:20 and 1:120 stoichiometry. At the same time, there was a decrease in potential energy, as indicated in the central panel of Figure 9. Regarding the competitive adsorption of (*M*)-5H and (*P*)-5H, the (*P*)-6H enantiomer molecules kinetically adsorbed faster than the (*M*)-6H molecule. During the MD simulation time at 300 K, the isomerization of these 5-aza[6]helicenes was not observed (see Table 2) as can be seen from the comparison of the Θ values reported in Table 1. Therefore, some changes were observed in terms of the value of the Θ dihedral angles of two enantiomers adsorbed near the minor groove and the major groove of the DNA fragment with respect to the Θ values calculated for the isolated single molecule of [6]helicenes.

**Table 2:** Values of Θ Dihedral Angles in the Optimized Geometries at the End of MD Run Considering the Enantiopure Compounds, (*M*)-6H (Negative Θ Values in Italic) and (*P*)-6H (Positive Θ Values), As Well As the Racemic Mixture at Small Concentration (20 Molecules of Helicene Interacting with the DNA Fragment)

(*M*)-HA Θ value (deg)	(*P*)-HA Θ value (deg)	racemic mixture Θ value (deg)
–34.845	28.906	–33.509
–34.042	32.411	29.6
–34.097	30.561	29.335
–34.309	30.143	–35.281
–34.571	29.894	–32.191
–29.006	32.999	–29.811
–30.403	30.907	–28.395
–35.449	33.004	–31.265
–27.265	33.139	27.465
–35.485	35.388	30.308
–29.408	30.426	34.277
–31.562	29.24	30.72
–32.144	29.051	–32.22
–31.986	32.635	–31.172
–32.355	31.983	–29.936
–32.067	29.356	31.234
–29.843	29.356	29.065
–31.724	34.295	30.204
–28.727	32.277	–33.123
–28.275	30.206	32.905

**Figure 10:**
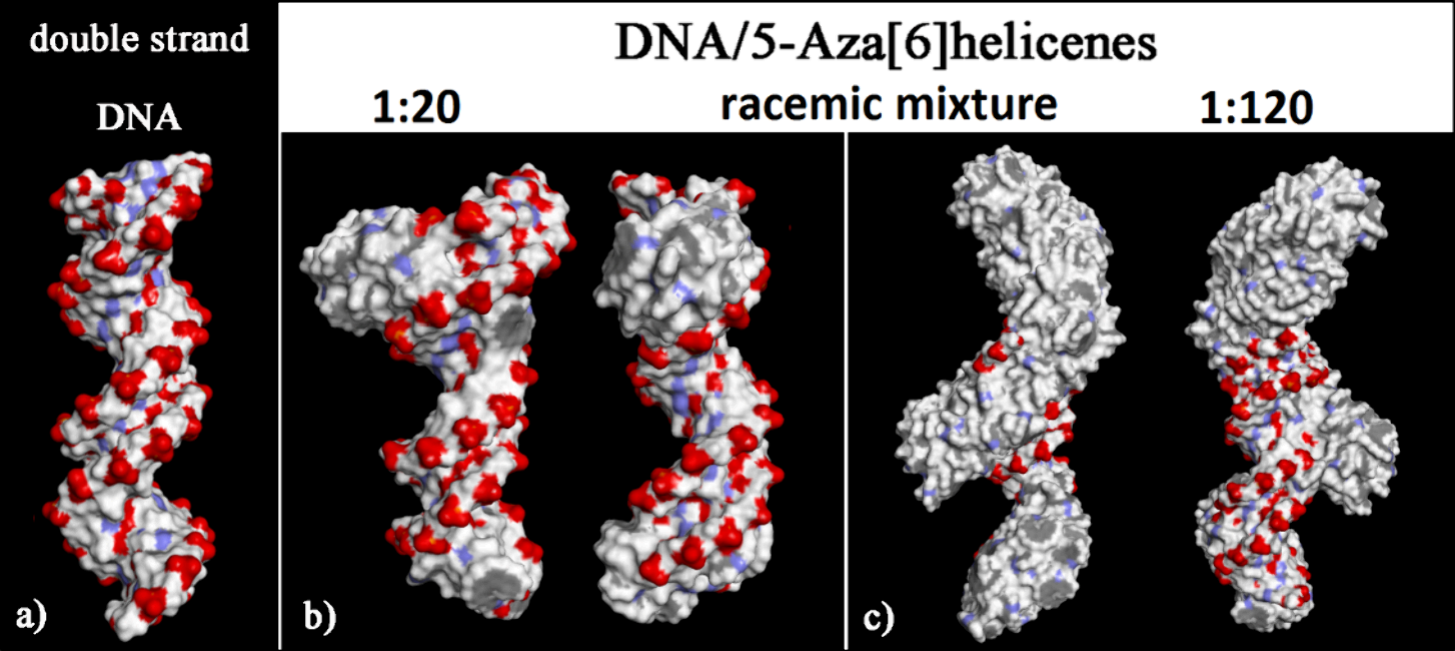
Solvent-accessible surface area colored by the atom charge for the optimized geometries of double-stranded DNA from data from the Protein Data Bank on the left (panel a) of the final optimized geometries of racemic mixtures at a small concentration with (*M*)-6H/(*P*)-6H in 10:10 stoichiometry in panel (b) and at a larger concentration with (*M*)-6H/(*P*)-6H in 60:60 stoichiometry on the right in panel (c). The color code is the same as in Figure 6.

At higher concentrations (see panel c in Figure 10), the adsorption process occurred preferentially in the major groove and, subsequently, during the process of self-aggregation of the helicene molecules. In racemic mixtures, a more ordered adsorption process was observed at larger concentrations and with ordered aggregation, not only at the end of the finite DNA structure but also following the DNA structure.

The SASA of the optimized geometries after the MD run is shown in Figure 10. Adsorbed and self-aggregated helicenes on chiral DNA architecture at higher concentrations exhibited a hydrophobic helicoidal coating exposed to the biological environment, especially following the major groove.

**Figure 11:**
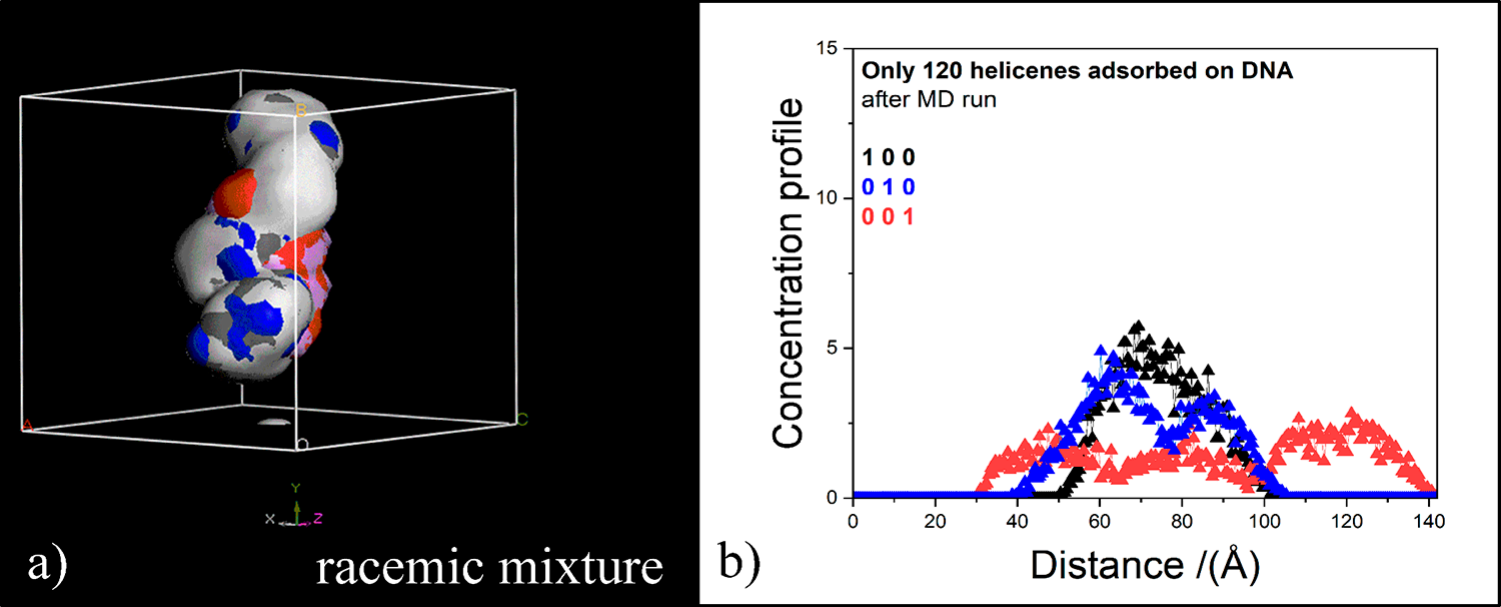
In panel (a) density field representation of the optimized final geometry of the higher concentration racemic mixture with (*M*)-6H/(*P*)-6H in 60:60 stoichiometry adsorbed on DNA structure, as in panel (c) of Figure 10, is shown. The color code is the same as in Figure 6. The atomic density concentration profile of the only helicene molecules within evenly spaced slices parallel to the *bc*, *ca*, and *ab* planes are reported in black, in blue, and in red line symbols in panel (b).

In Figure S6, the side view and top view of the only helicenes adsorbed on the major groove of the chiral DNA structure in the final optimized geometries considering 120 (*M*)-6H and (*P*)-6H enantiomers on the left (panel a) and on the right (panel b), respectively, without DNA for clarity, are shown. The (*M*)-6H enantiomers are colored in red and the (*P*)-6H enantiomers are colored in blue. The (*P*)-6H enantiomers are continuously adsorbed along the line defined by the major groove, and the (*M*)-6H enantiomer also follows the helical groove. The concentration profile of only the helicenes in racemic mixture adsorbed on the DNA structure as in the geometry shown in panel (c) of Figure 10, with the important details of the Cartesian axes in panel (a) of Figure 11, is reported in panel (b) of Figure 11. The helicenes are adsorbed on the DNA structure, following the orientation of the double helix.

## 4 Conclusions

Using MM/MD simulations, the adsorption process on DNA architecture and the possible self-aggregation process of chiral [5]-aza[6]helicenes at different concentrations were studied. Helicenes adsorb in two ways, either by following the DNA chiral surface or by forming aggregates on the DNA surface. Sometimes these aggregates are very similar to those formed in enantiopure compounds or racemic mixtures without DNA in a simulation box. Helicenes display local ordering as in liquid crystal systems as well as an orientational order at long-range near the DNA surface following its helicoidal structure. The formed aggregates attached to the DNA surface are very stable during simulation time, as found in a previous study^30^ considering [5]-aza[5]helicene molecules. The positional and orientational order of helicenes on the DNA surface in the final structure is interesting. Without DNA, different arrangements are possible and equally stable. After adsorption on DNA, small conformational changes take place. It is interesting how the simultaneous presence of different chiral molecules in the racemic mixture influences the kinetics of the adsorption process. [5]-Aza[*n*]helicenes with five or six aromatic rings adsorbed on double-stranded B-DNA modified the hydrophilicity of the DNA exposed to the biological environment. These new stable adsorbed architectures likely interact differently with light. The chiral structure of DNA acts as a nucleation substrate for the self-aggregation process of helicenes. The structure of DNA appears to be a chiral model. The helicenes self-aggregate on a fixed structure in these simulations. In future work, the DNA surface will have freedom of motion to better understand the possible intercalation process and whether the DNA architecture can be stabilized by a stable coating formed by chiral molecules such as helicenes.

The helix pitch (height of a turn) of A-DNA is 28.6 Å. The diameter of A-DNA is 20 to 25% shorter than that of B-DNA due to the smaller rise per turn. It would be interesting to study the interaction of the helicenes adsorbed on different forms of DNA, differing based on the curvature exposed to the biological environment. The change in helix pitch and in the geometry of the grooves can probably induce different adsorption geometries and therefore different interaction energies and therefore possibly different light emissions. Using the same methodology reported here, MM and MD methods can be useful tools to investigate at the atomistic level some similarities and some differences, just as found in my previous work on single-walled carbon nanotubes inducing different adsorption on protein fragments adsorbed in their outer surface or inner cavities according to their curvature.^33^ The classic macroscopic representation of a nematic mesophase is a herd of sheep walking along a mountain path. The two DNA grooves are akin to the mountain path but with important differences: they are not flat, they are curved grooves, and they exhibit chiral discrimination. They are an interesting substrate for selective adsorption, dictating the direction of the helix winding, with a specific pitch, inducing the formation of more ordered mesophases than a nematic mesophase with possible interaction with light of a suitable wavelength.

The basic idea is to predict, at an atomistic level, the ordered shape imprinted on the helicenes adsorbed on a chiral surface, such as DNA, just as it is possible to predict, at a macroscopic level, the shape of bronze in statues formed by casts in lost-wax casting, with the detail of a smile, a muscle, and beard filaments as in Greek and Roman statues. A coating of molecules with optical properties would have a dual role. Outside the helicenes can interact with light and show us if geometrically ordered and stable from an energy point of view, their color and shape, while in direct contact with the surface, they can act as a mold of the surface itself, B-DNA, Z-DNA, A-DNA, or other chiral surfaces.

## Supporting Information Available

All initial nonoptimized geometries and animations of the MD runs discussed; the optimized geometry of the helicenes adsorbed on the chiral DNA structure considering 120 (*M*)-6H and (*P*)-6H enantiomers without DNA for clarity reported together with all θ dihedral angle values calculated for all molecules of the first layer closer to the DNA chiral structure (PDF)
The MD runs obtained starting from the four different initial optimized geometries considered are reported in file.avi generated by using Materials Studio program, visualizing every 10 frames of the 1000 frames saved during MD runs lasting 20 ns (ZIP)

## Abbreviations

B-DNA: right-handed double-helical structure of DNA
MM: molecular mechanics
MD: molecular dynamics
(*M*)-5H: (*M*)-5-aza[5]helicene
(*P*)-5H: (*P*)-5-aza[5]helicene
(*M*)-6H: (*M*)-5-aza[6]helicene
(*P*)-6H: (*P*)-5-aza[6]helicene
CNT: carbon nanotube
PBC: periodic boundary conditions
NVT: number of particles, volume and temperature constant during the MD run
