# Supplementary material for: Adsorption and Self-Aggregation of Chiral [5]-Aza[6]helicenes on DNA Architecture: A Molecular Dynamics Study
Source: J Phys Chem B. 2023 Sep 26;127(39):8285–95. doi: 10.1021/acs.jpcb.3c02487 (PMC10561140; doi:10.1021/acs.jpcb.3c02487)
Supplement: Supplementary file 1 — jp3c02487_si_001.pdf [file jp3c02487_si_001.pdf]

Supplementary Materials

Adsorption and self-aggregation of chiral [5]-Aza[6]helicenes on DNA architecture: a molecular dynamics study

Giuseppina Raffaini *

[1] Department of Chemistry, Materials, and Chemical Engineering "Giulio Natta", Politecnico di Milano, Piazza L. Da Vinci 32, 20131 Milano (Italy); giuseppina.raffaini@polimi.it
[2] INSTM, National Consortium of Materials Science and Technology, Local Unit Politecnico di Milano, Milano – Italy.
* Correspondence: giuseppina.raffaini@polimi.it

Received: date; Accepted: date; Published: date

Figure S1. Initial two different non-optimized geometries considered for the 20 and 120 (*M*)-5-aza[6]helicene molecules on the left (panel a) and on the right (panel b), respectively, without DNA in the simulation box. The same geometries not reported here are also considered for the (*P*)-5-aza[6]helicene enantiomer. S3

The MD runs obtained starting from the four different initial optimized geometries considered are reported in file.avi generated by using Materials Studio program, visualizing every 10 frames of the 1000 frames saved during MD runs lasting 20 ns. S3

Figure S2. Initial two different non-optimized geometries considering racemic mixtures of 20 and 120 (*M*)-5-aza[6]helicene and (*P*)-5-aza[6]helicene enantiomers on the left (panel a) and on the right (panel b), respectively, without DNA in the simulation box. S4

The MD runs obtained starting from the two different initial optimized geometries considered are reported in file.avi generated by using Materials Studio program, visualizing every 10 frames of the 1000 frames saved during MD runs lasting 20 ns. S4

Figure S3. Initial two different non-optimized geometries considered for the (*M*)-5-aza[6]helicene molecules on the left near the DNA minor groove (panel a) and on the right near DNA major groove (panel b). The same geometries not reported here are also considered for the (*P*)-5-aza[6]helicene enantiomer. S5

The MD runs obtained starting from the four different initial optimized geometries considered are reported in file.avi generated by using Materials Studio program, visualizing every 10 frames of the 1000 frames saved during MD runs lasting 20 ns. S5

Figure S4. Initial two different non-optimized geometries considering 20 and 120 (*M*)-5-aza[6]helicene molecules on the left (see panel a) and on the right (see panel b), respectively, with DNA in the simulation box. The same geometries not reported here are also considered for the (*P*)-5-aza[6]helicene enantiomer. S6

The MD runs obtained starting from the four different initial optimized geometries considered are reported in file.avi generated by using Materials Studio program, visualizing every 10 frames of the 1000 frames saved during MD runs lasting 20 ns. S6

Figure S5. Initial two different non-optimized geometries considering 20 and 120 (*M*)-5-aza[6]helicene and (*P*)-5-aza[6]helicene enantiomers on the left (see panel a) and on the right (see panel b), respectively, with DNA in the simulation box. S7

The MD runs obtained starting from the two different initial optimized geometries considered are reported in file.avi generated by using Materials Studio program, visualizing every 10 frames of the 1000 frames saved during MD runs lasting 20 ns. S7

Figure S6. Side view and top view of the only helicenes adsorbed on the chiral DNA structure in the final optimized geometries considering 120 (*M*)-6H and (*P*)-6H enantiomers on the left (panel a) and on the right (panel b), respectively, without DNA for clarity. The (*M*)-6H enantiomers are colored in red, and the (*P*)-6H enantiomers are colored in blue. All θ dihedral angles values calculated for all molecules of the first layer closer to the DNA chiral structure are shown in light blue. S8

Information about Concentration profile. S9

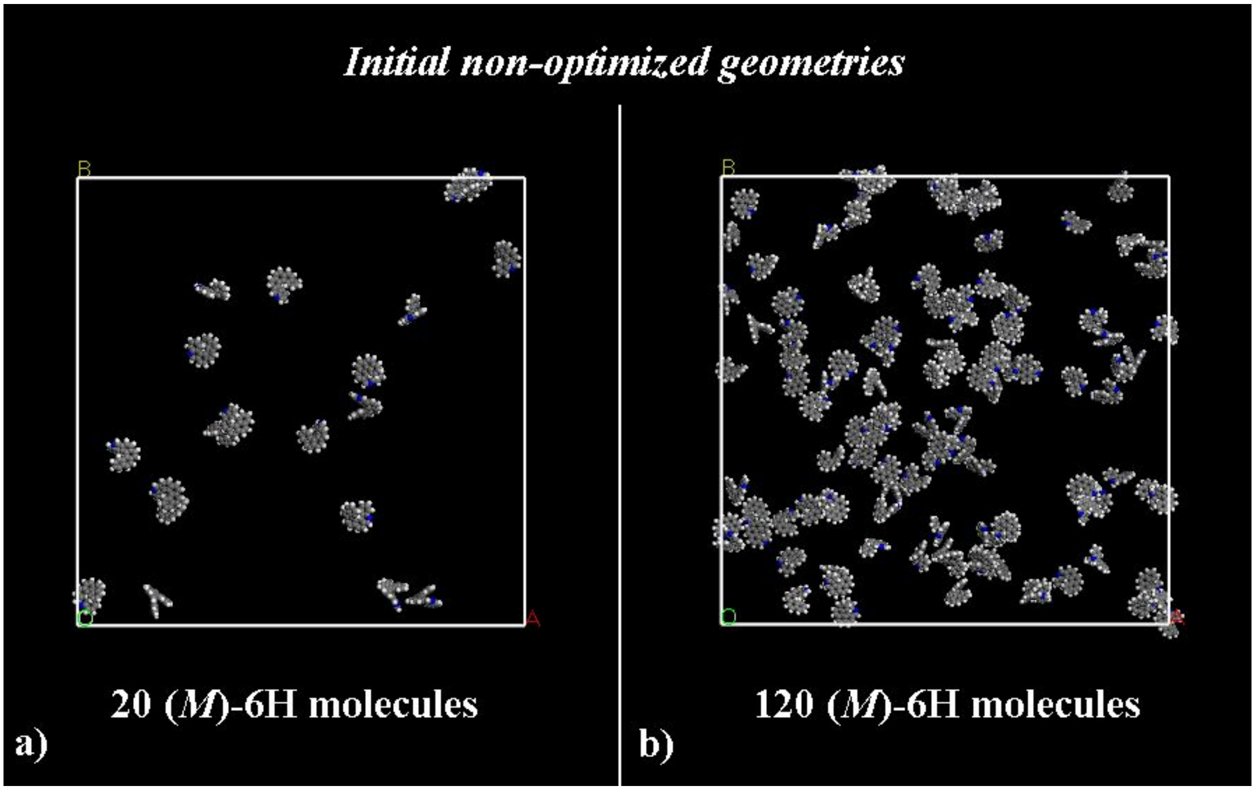

Figure S1. Initial two different non-optimized geometries considered for the 20 and 120 (*M*)-5-aza[6]helicene molecules on the left (panel a) and on the right (panel b), respectively, without DNA in the simulation box. The same geometries not reported here are also considered for the (*P*)-5-aza[6]helicene enantiomer.

The carbon atoms are in grey, the nitrogen atoms are in blue and the hydrogen atoms are in white. S3

The MD runs obtained starting from the four different initial optimized geometries considered are reported in file.avi generated by using Materials Studio program, visualizing every 10 frames of the 1000 frames saved during MD runs lasting 20 ns.

only_M_20_without DNA\MD run_only_M_20molecules_5-6H_20ns.avi
only_M_120_without DNA\MD run_only_M_120molecules_5-6H_20ns.avi
only_P_20_without DNA\MD run_only_P_20molecules_5-6H_20ns.avi
only_P_120_without DNA\MD run_only_P_120molecules_5-6H_20ns.avi

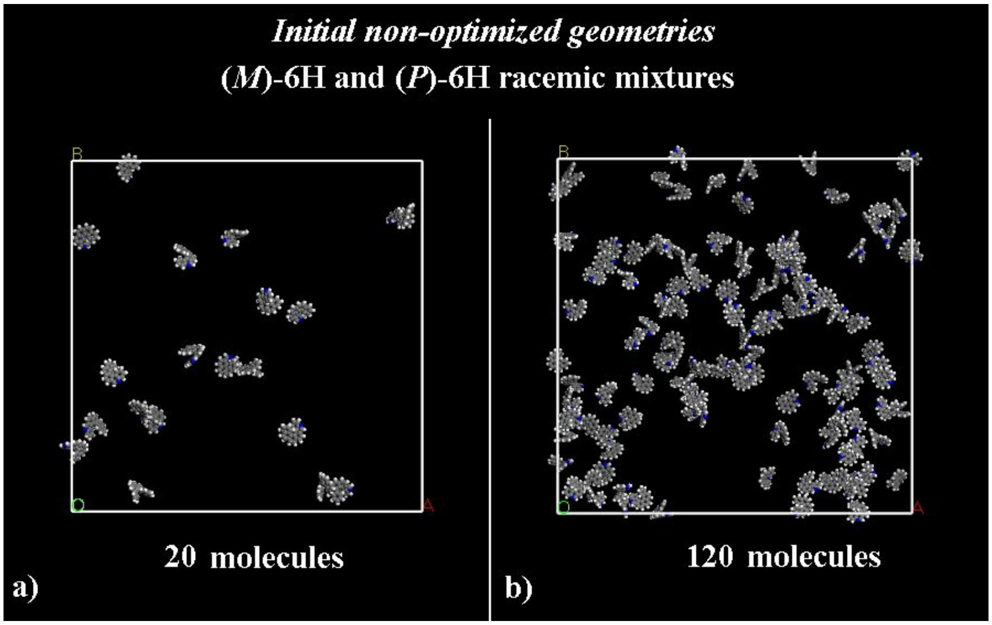

Figure S2. Initial two different non-optimized geometries considering racemic mixtures of 20 and 120 (*M*)-5-aza[6]helicene and (*P*)-5-aza[6]helicene enantiomers on the left (panel a) and on the right (panel b), respectively, without DNA in the simulation box.

The carbon atoms are in grey, the nitrogen atoms are in blue, and the hydrogen atoms are in white. S4

The MD runs obtained starting from the two different initial optimized geometries considered are reported in file.avi generated by using Materials Studio program, visualizing every 10 frames of the 1000 frames saved during MD runs lasting 20 ns.

MIX_only 20\MD run_only20m_10e10_azaMeP_20ns.avi

MIX_only_120\MD run_onlyMIX60e60_aza6_20ns.avi

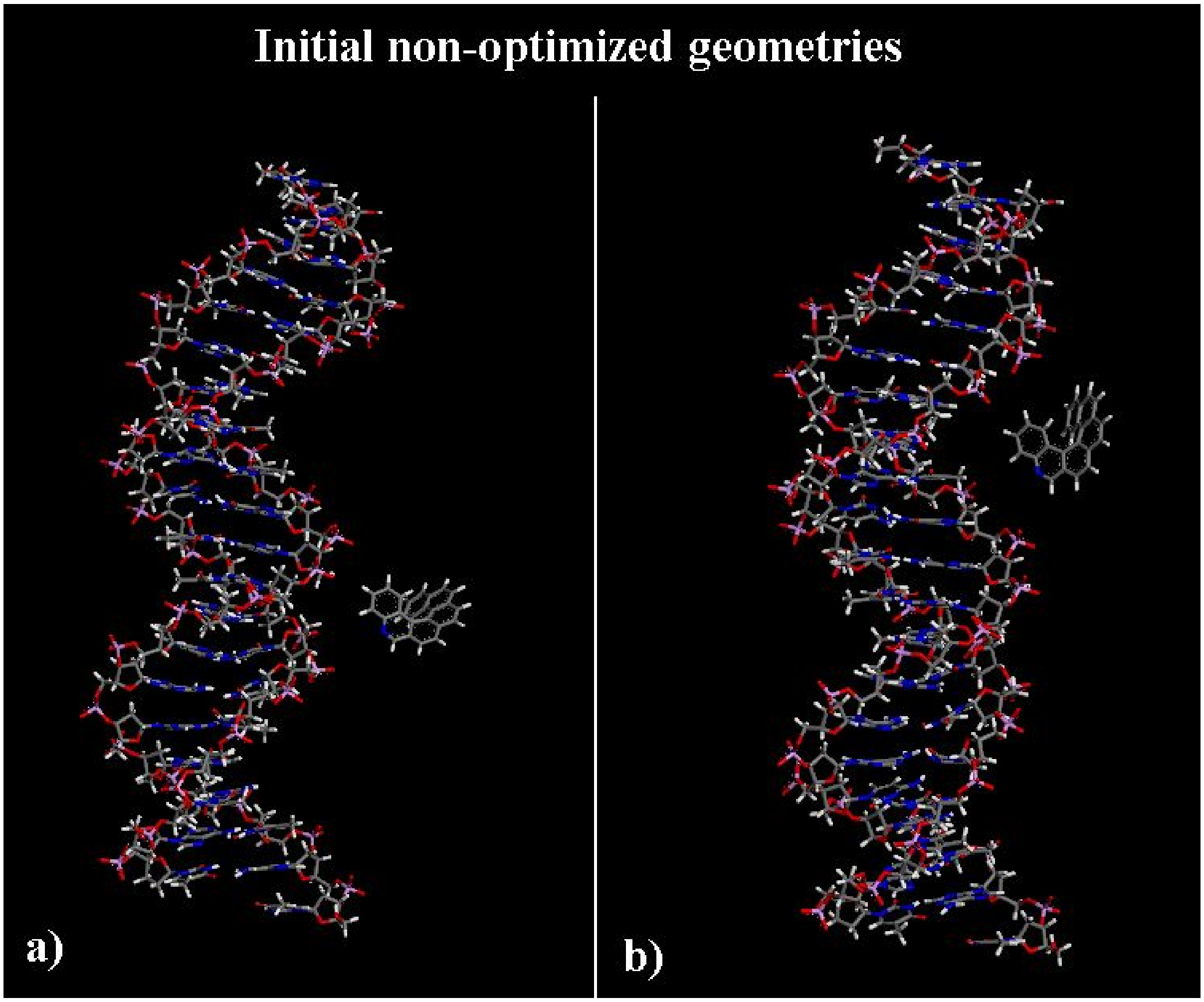

Figure S3. Initial two different non-optimized geometries considered for the (*M*)-5-aza[6]helicene molecules on the left near the DNA minor groove (panel a) and on the right near DNA major groove (panel b). The same geometries not reported here are also considered for the (*P*)-5-aza[6]helicene enantiomer.

The carbon atoms are in grey, the nitrogen atoms are in blue, the oxygen atoms are in red, the phosphorus atoms are in pink, and the hydrogen atoms are in white. S5

The MD runs obtained starting from the four different initial optimized geometries considered are reported in file.avi generated by using Materials Studio program, visualizing every 10 frames of the 1000 frames saved during MD runs lasting 20 ns.

M_pos1\MD run_DNA_M_5-6H_single molecles_pos_1_minorGROVE20ns.avi
M_pos2\MD run_DNA_M_5-6H_single molecles_pos_2_majorGROVE_20ns.avi
P_pos1\MD run_DNA_P_5-6H_single molecles_pos_1_minorGROVE20ns.avi
P_pos2\MD run_DNA_P_5-6H_single molecles_pos_2_majorGROVE_20ns.avi

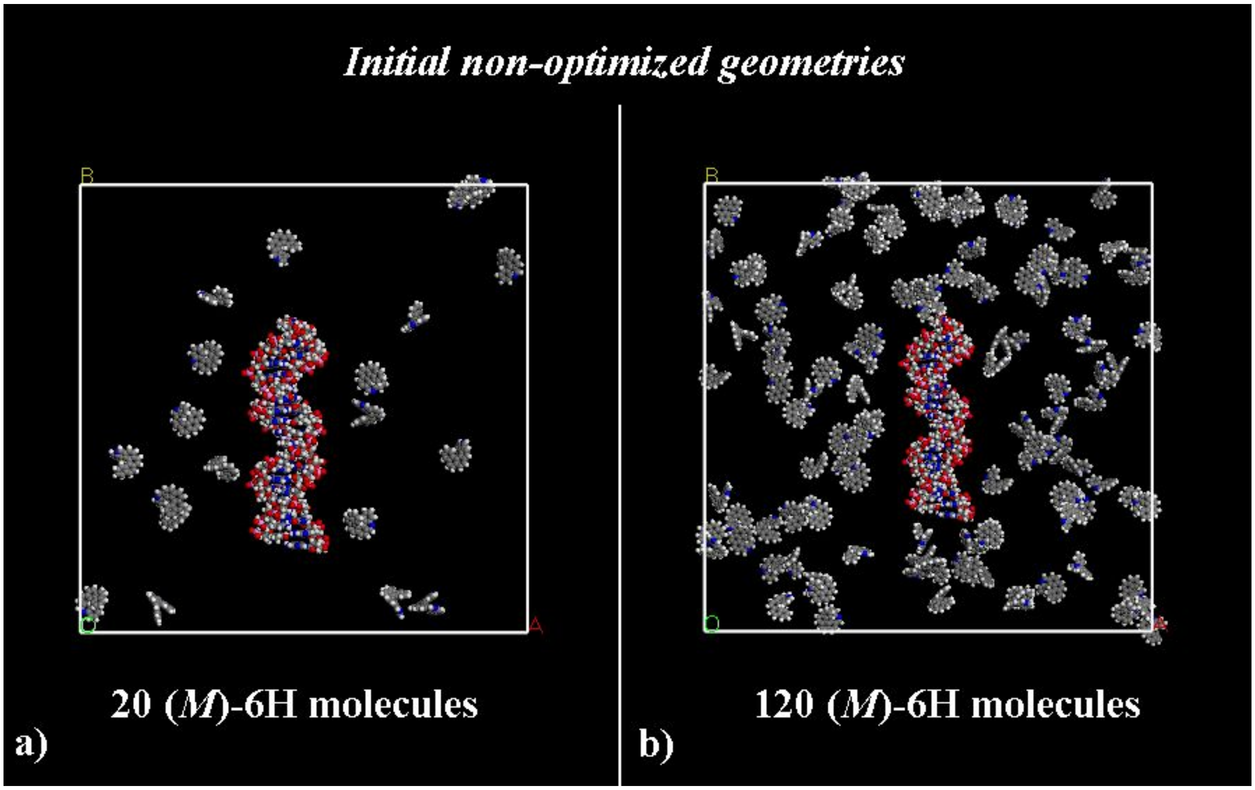

Figure S4. Initial two different non-optimized geometries considering 20 and 120 (*M*)-5-aza[6]helicene molecules on the left (see panel a) and on the right (see panel b), respectively, with DNA in the simulation box. The same geometries not reported here are also considered for the (*P*)-5-aza[6]helicene enantiomer.

The carbon atoms are in grey, the nitrogen atoms are in blue, the oxygen atoms are in red, the phosphorus atoms are in pink and the hydrogen atoms are in white. S6

The MD runs obtained starting from the four different initial optimized geometries considered are reported in file.avi generated by using Materials Studio program, visualizing every 10 frames of the 1000 frames saved during MD runs lasting 20 ns.

DNA_M_20\MD run_DNA_M_20molecules_5-6H_20ns_CPK.avi

DNA_M_120\MD run_DNA_M_120molecules_5-6H_20ns_XY.avi

DNA_P_20\MD run_DNA_P_20molecules_5-6H_20ns.avi

DNA_P_120\MD run_DNA_P_120molecules_5-6H_20ns_XY_lato.avi

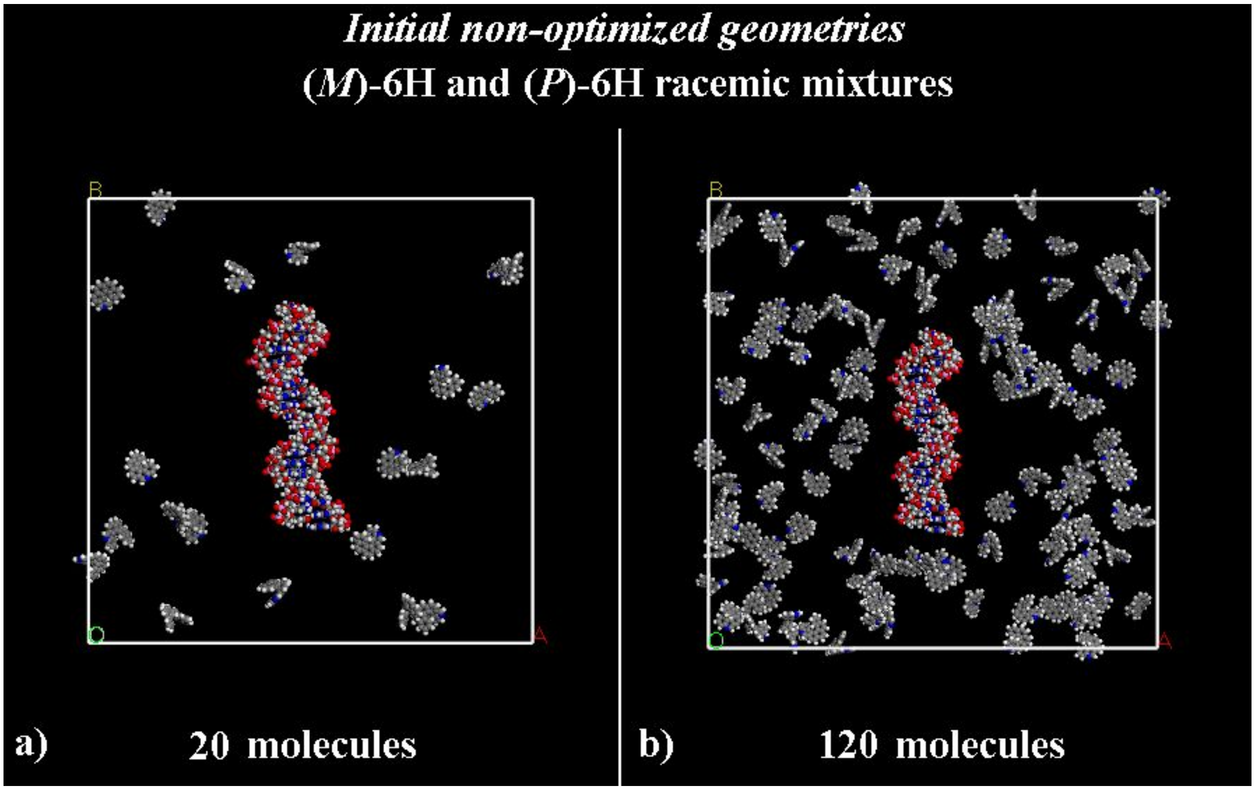

Figure S5. Initial two different non-optimized geometries considering 20 and 120 (*M*)-5-aza[6]helicene and (*P*)-5-aza[6]helicene enantiomers on the left (see panel a) and on the right (see panel b), respectively, with DNA in the simulation box.

The carbon atoms are in grey, the nitrogen atoms are in blue, the oxygen atoms are in red, the phosphorus in pink and the hydrogen atoms are in white. S7

The MD runs obtained starting from the two different initial optimized geometries considered are reported in file.avi generated by using Materials Studio program, visualizing every 10 frames of the 1000 frames saved during MD runs lasting 20 ns.

MIX_DNA_20\MD run_cella_DNA_mix_10e10_6_20ns_CPK.avi

MIX_DNA_120\MD run_cellaDNAmix_aza6_120_20ns_XY.avi

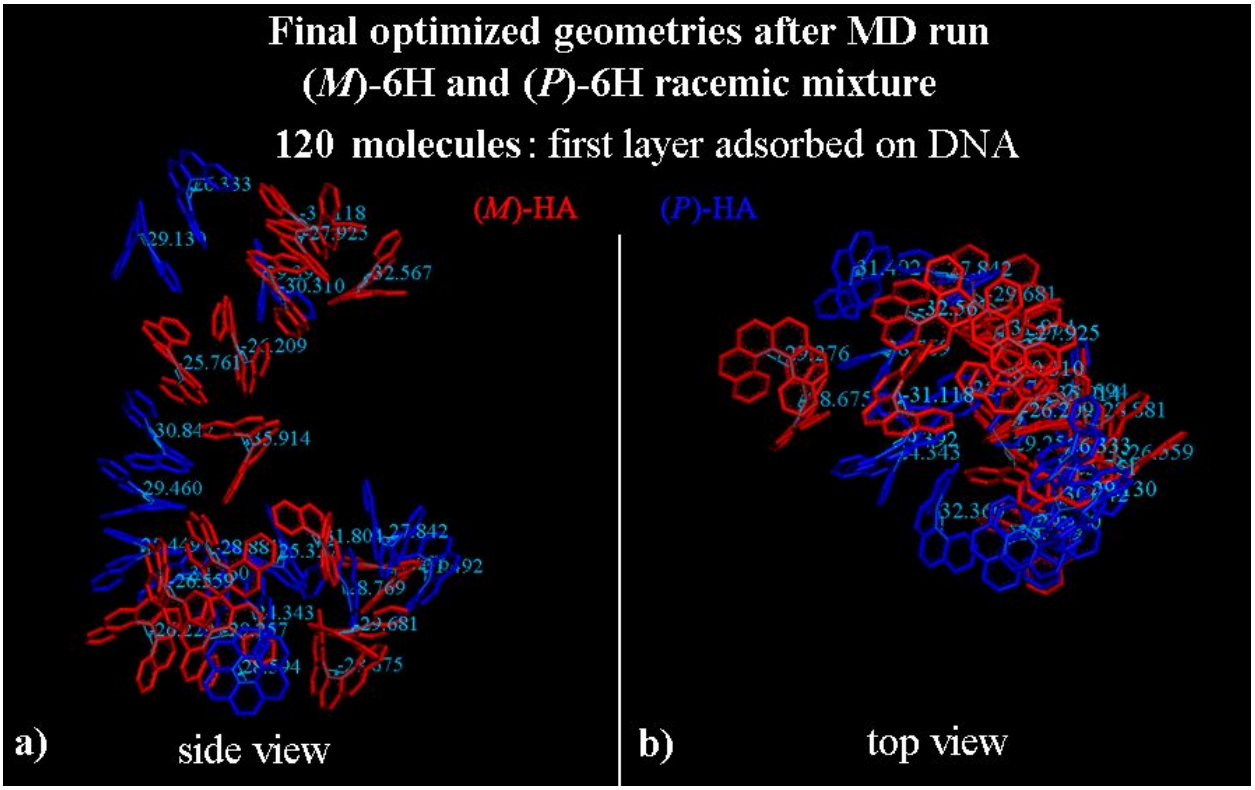

Figure S6. Side view and top view of the only helicenes adsorbed on the chiral DNA structure in the final optimized geometries considering 120 (*M*)-6H and (*P*)-6H enantiomers on the left (panel a) and on the right (panel b), respectively, without DNA for clarity. The (*M*)-6H enantiomers are colored in red, and the (*P*)-6H enantiomers are colored in blue. All θ dihedral angles values calculated for all molecules of the first layer closer to the DNA chiral structure are shown in light blue. S8

Concentration profile

The Concentration profile is calculated for 3D periodic structures by computing the profile of atom density within evenly spaced slices parallel to the bc, ca, and ab planes. In practice, this is equivalent to taking the a, b, and c components of the fractional coordinates of each atom and independently generating a plot for each component. For 2D periodic structures, the u and v components of the fractional coordinates are used, resulting in two plots.

As an alternative to generating profiles along each axis, it is also possible to specify a particular direction in the form of an (h k l) vector. In this case, Forcite will produce a single plot of the concentration profile parallel to the plane defined by that vector.

Each direction is divided equally into a number of bins (potentially, a different number for each axis). For each atom in the unit cell, a contribution is added into the relevant cell in the binning vector corresponding to each axis. The contribution is a constant value determined so that the average value along each axis becomes 1.0 (i.e., a homogeneous structure would have values close to 1.0).

The relative concentration of a set of atoms in a slab is:

relative [set]slab = [set]slab/[set]bulk

where

[set]slab = (no. atoms in slab)/(volume of slab)
[set]bulk = (total no. atoms in system)/(volume of system)

The relative concentration is a dimensionless quantity, a value of 2 means that there are twice the number of atoms in the slab that if all atoms were distributed homogeneously across the system. The total number of atoms across all slabs is equal to the number of atoms in the entire system. So the sum of the relative concentrations of all slabs is equal to the number of slabs.

For plotting, the bin values are converted from fractional positions to Cartesian values. To allow for the possibility of the unit cell changing, the values used for the unit cell dimensions are the average over all the frames under consideration.

As with other structural analyses, the concentration profile can be calculated for a subset of the atoms in the input structure by specifying a set in the input document or simply by manually selecting the atoms you wish to include in the calculation.
